# Assessment of vitamin status;  A, E and D in Egyptian neonates with IUGR: a cross sectional study

**DOI:** 10.1186/s12887-024-04624-2

**Published:** 2024-02-28

**Authors:** Hanaa Reyad Abdallah, Abderahman A. Abdelrazek, Eman Refaat Youness, Hisham A. Orban, Marwa A. Mahmoud, Ahmed Helal El Sayed, Moushira Zaki

**Affiliations:** 1 Biological Anthropology Department, Medical Research and Clinical Studies Institute, National Research Centre, Cairo, Egypt; 2 Department of Pediatrics, Faculty of Medicine, Cairo University, Cairo, Egypt; 3 Medical Biochemistry Department, Medical Research and Clinical Studies Institute, National Research Centre, Cairo, Egypt; 4 Department of Pediatrics, Faculty of Medicine for boys, Al- Azhar University, Cairo, Egypt

**Keywords:** Intrauterine growth retardation (IUGR), Vitamin status, Vitamin A, Vitamin E, Vitamin D

## Abstract

**Background:**

Neonates with intrauterine growth retardation (IUGR) may present with fatal complications and permanent serious consequences. Vitamin status may influence fetal development. In this study we assessed vitamin A, E and D concentrations in umbilical cord blood in newborns with IUGR.

**Methods:**

Maternal data were obtained. Neonatal assessment included; age of gestation calculated from last menstrual period, Ultrasound (U/S), new Ballard, Apgar scores and anthropometric measurements including; Head circumference, length and weight. WHO growth percentile curves were used. Vitamin A, E and D in cord blood samples were measured by high performance liquid chromatography (HPLC) and ELISA consecutively.

**Results:**

A total of 86 full term newborns were enrolled in this study, 42 (48.8%) with IUGR with gestational age (33.59 ± 1.20) week by U/S and 44 (51.2%) appropriate for gestational age neonates with gestational age (38.70 ± 1.50). Ballard and Apgar scores (*p* < 0.05) and Z scores for weight, length and head circumference (*p* < 0.001) at birth were significantly lower in neonates with Intrauterine growth retardation (IUGR) than appropriate for gestational age (AGA) neonates. The levels of Vitamin A, E and D were significantly lower in the IUGR group than the AGA (*p* < 0.05) for all. Significant positive correlations of weight with vitamin A, and E cord blood levels were found (*p* < 0.05), while length was significantly positively correlated only with vitamin A (*p* < 0.05). Head circumference showed significant positive correlations with the three vitamins (*p* < 0.05) for all.

**Conclusion:**

Neonates with IUGR had significantly lower levels of Vitamin A, E and D than AGA neonates. Significant positive correlations of weight with vitamin A, and E cord blood levels was detected, while neonatal length was associated only with vitamin A level. The present study highlights the significance of nutritional policies for inhibiting deficiency of these vitamins during pregnancy and childhood.

## Background

Intrauterine growth restriction (IUGR) usually refers to poor fetal growth with a birth weight less than the 10th percentile for gestational age and sex [1, 2]. Neonates with IUGR are more susceptible to perinatal illness and death. In addition to metabolic and cardiac disorders at adult life [3]. Globally 7–15% of neonates are born with IUGR. Whereas, in developing countries, the prevalence rises to 30% – comprising Egypt – forming 50–60% of low birth weight (LBW) newborns (< 2500 gm) [4].

The definition of intrauterine growth restriction or small-for-gestational-age (SGA) is known as weight or crown-heel length below the 10th percentile for gestational age and sex at birth. The two expressions are mutually in use [5]. Three types of IUGR are known; type I (Symmetrical IUGR), type II (Asymmetrical IUGR) and type III (Type I + Type II). Type I is commonly produced by disorder in fetal cells proliferation, Type II and III are predisposed by external causes as deficiency of vitamins leading to impairment of growth after the 2nd trimester [6].

Vitamin A is a necessary micronutrient for preservation and maturation of cells, vision, growth and development of children and immunity. Deficiency of vitamin A is prevalent in developing countries particularly in Africa and South-East Asia. It plays a role in mothers’ death and unfavorable pregnancy outcomes. Minor insufficiency might elevate respiratory and gastrointestinal infections in children, impair physical growth, delay bone formation and reduce the chance of surviving from severe diseases [7].

The WHO has declared the deficiency of vitamin A as “a public health problem” in more than 70 countries worldwide and harming around a 1/3 of all pediatric age group from 6 to 59 months. Currently, nearly 190 million children before school age and 20 million pregnant females are estimated to have deficiency of vitamin A [7].

Vitamin A from the mother is acquired by child in the course of pregnancy via the placenta and after birth through breast milk. Limited studies have sufficiently investigated the incidence of vitamin A deficiency in the IUGR neonates [8].

On the other hand, the passage of vitamin E through the placenta is restricted. Hence, neonates are predisposed to be deficient in vitamin E which could lead to its decrease in blood and tissues at birth [9]. Vitamin E deficiency may lead to restriction of fetal growth. This was hypothesized considering the fact that vitamin E is able to raise the production of prostaglandins I2 and E2, both lead to vasodilatation and may increase fetal blood supply [10]. Accordingly, reduced levels of vitamin E can subsequently affect the nutrients transfer to the fetus, decreasing its growth [11]. Nevertheless, although vitamin E was supposed to have a potential effect on fetal growth, yet, there are limited researches to assess this association taking weight and age of gestation into consideration.

Moreover, conferring to the Food and Agriculture Organization (FAO), there are no data on deficiency of vitamin E in Africa [12]. To the best of our knowledge, few studies were conducted evaluating Vitamin E condition in Egyptian pregnant women and their infants to assist adjust nutrition intervention in perinatal period and improve pregnancy outcomes in mothers and neonates. It is well documented that vitamin E concentrations, especially α-tocopherol, are linked with pregnancy outcomes, like fetal growth [13, 14]. Thus, investigating the relation between levels of vitamin E in blood with certain pregnancy outcomes like gestational duration, growth of fetus, and other maternal and fetal aspects would explore the necessity of dietary interferences to improve outcomes of pregnancy [15].

Another important vitamin which has a crucial role in fetal growth is vitamin D. However, different studies performed to associate the deficiency of vitamin D with Intrauterine Growth Retardation (IUGR) have revealed variable findings. Pérez-López et al., and Maugeri et al., suggested that IUGR may be attributed to the point that vitamin D in adequate levels is needed to keep sufficient levels of calcium and phosphate. Consequently, it permits the crucial mechanisms of bone mineralization and growth throughout fetal intrauterine period [16, 17]. The current study aimed to assess vitamin A (Retinol), E (alpha- tocopherol) and D (25 (OH) D) levels in the umbilical cord blood in newborns with intrauterine growth retardation (IUGR) and compare them with those in neonates with appropriate weight for gestational age (AGA), evaluating the nutrition of the two cohorts regarding these vitamins which can be adjusted variables impacting neonatal outcomes. As far as we know, the present study is the first one evaluating the vitamin status involving three vitamins; A, E and D in Egyptian neonates with IUGR.

## Methods

### The study design

This study was a cross sectional observational study.

### Participants

Cases were collected from Al-Azhar University Hospital and laboratory investigations were done in the National Research Centre.- The study was conducted on 86 neonates divided into 2 groups:

- Group 1 (intrauterine growth restriction group): included 42 neonates of both sexes born with gestational age (37–41) week as estimated by the last menstrual period with intrauterine growth restriction (IUGR); as diagnosed by gestational weight below the 10th percentile.
- Group 2 (appropriate for gestational age group): comprised 44 appropriate for gestational age neonates of the same gestational age (37–41 weeks), sex matched, born for women with normal blood pressure and without any medical or obstetrical complications.

In both groups the neonates were delivered with normal vaginal delivery. Neonates born with caesarean section (CS) or assisted vaginal delivery or those born for mothers with known medical conditions affecting placental sufficiency; e.g. diabetes, hypertension and pre-eclampsia were excluded. Neonates born with congenital anomalies were also excluded from the study.

### Procedure

Clinical outcomes of neonates were defined as gestational age at delivery, low APGAR scores (< 7) at 1 and 5 min (APGARs are universally assigned and are a standardized way of describing an infant’s stability as they transit from the intrauterine to extra-uterine environment by scoring heart rate, respiratory effort, tone, and oxygenation, making this a good marker to use between these populations), as well as the birth growth parameters of length, weight, and head circumference (dichotomized at the 10th percentile for each parameter), and, weight-for-age, length-for-age, and head-circumference-for-age (dichotomized at a z-score). Z-scores were calculated using the WHO infant growth parameter standards [18].

Thus, all neonates of both groups were subjected to the following:

Full maternal data: were taken including age of mother, gravidity, parity, abortions, gestational age by early ultrasound and maternal illnesses as pre-eclampsia, hypertension, gestational diabetes, premature rupture of membrane. Obstetric data including mode of delivery and gestational age at delivery by ultrasound were also obtained.

Neonatal assessment: including gestational age by new Ballard, sex of neonate, Apgar score at 1 and 5 min and full general and systemic examinations of the newborns.

Growth analysis: Intrauterine growth was evaluated using the anthropometric indices of birth weight and length at birth by gestational age, Body mass index (BMI) was calculated as weight (kg)/height (m2). Head circumference was measured using non stretchable tape. Birth growth parameter Z-scores were calculated using the World Health Organization (WHO) Child Growth Parameter’s Anthro software for SPSS (SPSS Inc., Chicago, IL, USA). All measures were performed using standardized equipment and following the recommendations of the International Biological Program. Newborns were classified as intrauterine growth retardation neonates (IUGR) when their percentiles were < 10, appropriate for gestational age (AGA) when their percentiles were 10 to 90 [19, 20].

All the newborns were born at term. Gestational age was assessed from the date of last menstrual period and concurrent clinical assessments were done using the New Ballard score [21].

### Biochemical investigations

Sample collection and storage: A 5 ml of blood were taken from the umbilical cords of the neonates in the delivery room. Blood was allowed to clot for 30 min at 25 °C. It was then centrifuged at 3,000 rpm for 15 min at 4 °C, and the serum were separated into clean properly labeled tubes and freezed at -80 °C until the following investigations were done:

#### Analysis of vitamins A and E by high performance liquid chromatography (HPLC): was done as follows

Sample extraction:

One hundred µl of serum were mixed with ethanol. The micronutrients were extracted from the aqueous phase in hexane and dried under vacuum. The extract was re-dissolved in ethanol and acetonitrile and filtered to remove any insoluble materials.

HPLC condition for vitamin A:

Twenty µl of the filtrate were injected onto a C18 reversed phase column ( 25 cm×10.00 mm, 5 μm particle size) and isocratically eluted with a mobile phase consisting of ethanol/acetonitrile 50:50 with 0.1% trimethylamine, and were delivered at a flow rate of 1 ml/min. UV detection was performed at 325 nm. Serial dilutions of standards were injected, and their peak areas were determined. A linear standard curve was constructed by plotting peak areas vs. the corresponding concentrations. The concentrations in samples were obtained from the curve.

HPLC condition for vitamin E:

Twenty µl of the filtrate were injected onto a C18 reversed phase column (15 cm×10.00 mm, 5 μm particle size) and the thermostat was adjusted to 30ºC with a mobile phase consisting of 100% methanol delivered at a flow rate of 1 ml/min. Fluorescence detector was used and performed at 295 and 330 (excitation and emission). Serial dilutions of standards were injected, and their peak areas were determined. A linear standard curve was constructed by plotting peak areas vs. the corresponding concentrations. The concentrations in samples were obtained from the curve [22].

#### Assessment of vitamin D

(25 (OH) D) was assessed by vitamin D direct ELISA Kit (EIA-4696) (DRG ® International, Inc. USA) [23].

### Statistical analysis

The continuous variables were verified to be normal using the Kolmogorov-Smirnov test. Pearson’s correlation was used to verify the correlation between the levels of the three vitamins A, E, and D and the Z scores of weight, length and head circumference, since the data presented normal distribution. The Student’s t-test was used for comparison between the 2 groups. The data were analyzed in Statistical Package for the Social Sciences (SPSS), version 17.0. Significance level was *p* < 0.05.

## Results

A total of 86 newborns were enrolled in this study, 42 (48.8%) of them [22 females (52.4%) and 20 males] with IUGR with mean gestational age (33.59 ± 1.20) week as estimated by U/S at delivery time. The study also comprised 44 (51.2%) appropriate for gestational age neonates [23 females (52.3%) and 21 males] with mean gestational age (38.70 ± 1.50) by U/S at labour.

Regarding the maternal characteristics; there were no significant differences between both groups (*p* > 0.05) as shown in Table 1.

**Table 1:** Maternal characteristics in IUGR and AGA groups

Variable	Group	Mean ± SD	*P*
Maternal age	IUGR	27.10 ± 4.85	0.927
	AGA	27.12 ± 6.18	
Maternal gravity	IUGR	2.76 ± 1.66	0.232
	AGA	3.31 ± 2.44	
Maternal parity	IUGR	1.27 ± 1.38	0.505
	AGA	1.47 ± 1.46	
Maternal abortion	IUGR	0.54 ± 1.25	0.425
	AGA	0.85 ± 2.26	

IUGR: intrauterine growth retardation, AGA: appropriate for gestational age, Student’s T test, *p* < 0.05 = significant

As regards to the characteristics of the studied neonates, there was significant difference in GA diagnosed by U/S at delivery (*p* = 0.01), Ballard score and Apgar score (*p* < 0.05) between IUGR and AGA neonates.

Z scores for Weight, length and head circumference at birth were significantly different between the 2 groups (*p* < 0.001) for all (Table 2).

**Table 2:** Characteristics of the studied IUGR and AGA newborns

Parameter	IUGR group *N* = 42	AGA group *N* = 44	*P*
GA by LMP (weeks)	38.27 ± 1.15	38.66 ± 2.15	0.625
GA by US at delivery (weeks)	33.59 ± 1.20	38.70 ± 1.50	0.01
Ballard score	38.11 ± 1.03	38.84 ± 1.22	0.004
1 min Apgar score	6.84 ± 1.89	7.83 ± 0.61	0.002
5 min Apgar score	9.63 ± 0.82	9.89 ± 0.74	0.115
Z length	-3.59 ± 1.02	− 0.69 ± 1.27	0.000
Z weight	-2.70 ± 0.67	− 0.47 ± 1.15	0.000
Z BMI	-1.46 ± 1.01	− 0.19 ± 1.45	0.000
Z Head circumference	-1.89 ± 0.95	0.06 ± 1.22	0.000

GA: Gestational age, LMP: Last menstrual period, IUGR: Intrauterine growth retardation, AGA: Appropriate for gestational age, Z length: Z scores of length, Z weight: Z scores of Weight, Z BMI; Z scores of Body Mass Index, Z head circumference: Z scores of Head circumference, *p* < 0.05 = significant

Regarding the vitamin status, there were significant differences in the levels of Vitamin A, Vitamin E and Vitamin D in the umbilical cord blood between the two groups (*p* < 0.05) for all as shown in Table 3.

**Table 3:** Comparison of vitamins serum levels between IUGR and AGA newborns

Vitamin	IUGR	AGA	*P*
Vitamin A (umol/L)	1.13 ± 0.13	1.53 ± 0.05	0.0001
Vitamin E (ug/ml)	16.68 ± 3.27	22.59 ± 1.11	0.0001
Vitamin D (ng/ml)	23.67 ± 9.01	33.53 ± 7.65	0.008

Student’s T test, *P* < 0.05 = Significant

Regarding the correlation of the three vitamins status with the anthropometric parameters of the studied neonates; there were significant positive correlations of weight for age Z scores with vitamin A, and E cord blood levels (*p* < 0.05). While length for age Z scores were significantly positively correlated only with vitamin A cord blood level (*p* < 0.05). However, BMI for age Z scores didn’t show any significant correlations with any of the three vitamins cord blood levels. Meanwhile, head circumference Z scores showed significant positive correlations with the three vitamins cord blood levels (A, E, D) at birth (*p* < 0.05) for all, as present in Table 4.

**Table 4:** Correlation between cord blood levels of vitamins and anthropometric measurements of the total studied neonates

Anthropometric parameter	Vitamin A		Vitamin E		Vitamin D	
	r	*P*	R	*P*	R	*p*
Z Weight	0.506	0.019	0.530	0.020	0.353	0.083
Z Length	0.508	0.026	0.333	0.192	0.150	0.494
Z BMI	0.295	0.220	0.364	0.151	0.363	0.089
Z Head circumference	0.701	0.001	0.640	0.006	0.566	0.005

Pearson Correlation test, Z length: Z scores of length, Z weight: Z scores of Weight, Z BMI; Z scores of Body Mass Index, Z head circumference: Z scores of Head circumference, *p* < 0.05 = significant

However there were no significant correlations between the three vitamins and the Apgar scores of the neonates in our study (*p* > 0.05) as shown in Table 5.

**Table 5:** Correlation between vitamins serum levels and Apgar scores of the neonates with IUGR

Apgar score	Vitamin A		Vitamin E		Vitamin D	
	r	*P*	r	*P*	r	*P*
1 min Apgar score	0.152	0.500	0.033	0.891	0.185	0.366
5 min Apgar score	0.231	0.302	0.101	0.673	0.076	0.712

Pearson Correlation test; p < 0.05 = significant

## Discussion

Pregnant females in countries with low-and middle-income (LMICs) are liable to have growth retardation of fetus six times greater than those in countries with high-income [24]. Intrauterine growth retardation develops when nutritional provision to the fetus is affected, leading to redistribution of blood supply to important structures, such as the brain, and far from the rest of tissues [25]. Retardation of growth of fetus may be produced by: placental causes, like insufficiency of the placental or pre-eclampsia; mother causes, like chronic illness, serious malnourishment or multipara; or causes related to fetus, such as certain syndromes or chromosomal aberrations. Growth Retardation of fetus is accompanied with increased possibility of intrauterine fetal death, cardiac and kidney abnormalities and affection of nervous system and disorders of development [26].

Fat-soluble vitamins A, D, E have significant functions and effects on child growth and development. Vitamin A and vitamin D are included in calcium and phosphorus metabolism, normal embryogenesis, immunity, vision, gene expressions, and blood formation [27]. Their deficiency is responsible for recurrent infections, anemia, growth retardation, diminished vision, affection of bone growth, and demice [28].

Deficiency of vitamin A represents a serious pediatric health hazard in countries of low- and middle income [29]. Deficient Vitamin A can be injurious to development of the brain in fetus and in newborns can cause high susceptibility to bronchopulmonary dysplasia and increase the possibility of inflammation [30, 31].

As the major reason of IUGR is placental insufficiency, it was accompanied with prominent oxidative stress. There are reduction in genes expression responsible for the role of mitochondria in oxidative phosphorylation and elevated oxidative stress indicators in Placentas with IUGR [32]. Vitamin E is an antioxidant protects cells and counteracts damaging actions of free radicals. It regulates transduction of signals and expression of genes [33]. Thus, it is vital to ensure adequate fat-soluble vitamins concentrations for neonates [34].

Therefore, in this regard, the present study has been conducted to evaluate the vitamin condition in neonates with IUGR and revealed that; regarding vitamin A levels; they were significantly increased in cord blood of AGA neonates than IUGR group. Significant positive correlation of vitamin A cord blood level with fetal growth in the form of weight, length and head circumference Z scores of all the study population with was detected.

Our results disagree with Fernandes et al., study, who found that 18% of newborns were overweight and 8.2% had linear growth retardation (<-2 z-score). While in our study 48.8% of neonates were < − 2 z scores in length for age and sex. Regarding maternal characteristics in this study, the mean age of mothers in all the studied neonates was > than 20 y while in Fernandes et al., the age of 84.6% of mothers was ≥ 20 years [8]. On the other hand, maternal age of neonates with IUGR was statistically significantly less than that of AGA neonates (25.29 ± 4.55 years) in the study done by Chen et al., [35] which disagrees with our study as we didn’t detect any significant difference between mothers with IUGR neonates and those with AGA. The study done by Mohammad et al. reported that young (25.8 ± 2.1 years) age of the mother was risk factor for intrauterine growth restriction [36], which disagrees also with our results.

There is currently no agreement in the cut-off point for sufficient vitamin A concentration for newborns. Neonates typically have low serum vitamin A concentrations and low liver stores at birth. During the third trimester of pregnancy, the fetus starts to accumulate vitamin A and stores it in the liver, but the transfer from mother to child is limited. Usually, neonates are born with approximately half the vitamin A concentrations compared with their mothers [37]. The WHO does not currently recommend neonatal vitamin A supplementation as a public health intervention to reduce infant morbidity and mortality. At present, there are three suggestions to ensure neonatal vitamin A status, including improving maternal nutritional status through more frequent intake of vitamin A containing foods, promoting proper feeding for infants and young children, and reducing the burden of nutrient depleting infections [38].

In the current study; the mean serum vitamin A level among the IUGR neonates was 1.13 ± 0.13 versus 1.53 ± 0.05 umol/L in the AGA group. Whereas, in Fernandes et al., study; the mean level of vitamin A amongst all the studied newborns was 1.13 ± 0.60 µmol/L. However, on the contrary to the findings of our study, they didn’t find association between vitamin A level with weight or length in their study population [8].

On the other hand, Saunders et al., [39] reported insufficient levels of vitamin A in serum (< 1.05 µmol/L) among 45% of newborns, based on umbilical cord-blood samples. Meanwhile, Rondo et al. [40] found vitamin A deficiency (< 0.70 µmol/L) in serum of 14.6% of AGA newborns.

Vitamin A deficiency in neonates has been connected with low birth weight, even though a prior investigation proposed that it was unlikely to be a causal relation [41]. The results of the present study delineated positive correlations between cord blood vitamin A level and weight, length, and head circumference in our study population. However, no correlation was detected between vitamin A level and BMI.

Compatible with the results of the current study, Rondò et al. [42] proved that decreased concentrations of vitamin A had a great effect on growth and development. They detected the relation between vitamin A level of cord blood and anthropometric measures in 711 full term Brazilian neonates and found that vitamin A levels had significant correlation with birth weight, length, mid-upper arm, head and chest circumferences, and triceps skinfold thickness.

In study done by Liu et al., they found that vitamin A serum level positively correlated with neonatal weight at birth [34].

Additionally, parallel to our results, Pacifici, reported that elevated vitamin A cord blood concentrations raised neonatal birth weight and length thus increased the neonatal body size [43].

Moreover, Agarwal et al. [44] proved that significantly elevated concentrations of vitamin A in cord blood were seen with greater neonatal weight at birth and increasing gestational age and maturity. Their results showed that IUGR is related to deficiency of vitamin A.

Concerning vitamin E, despite its alleged role in enhancing growth of the fetus by increasing blood flow and subsequently, fetal nutrition throughout pregnancy, currently, no agreement is present regarding the association of its umbilical cord level with fetal growth [10].

In this context, we found that vitamin E (alpha-tocopherol) levels in our study neonates were significantly higher in the cord blood of the AGA group than the IUGR group. There were significant positive correlations of weight and Head circumference Z scores with vitamin E cord blood levels at birth for both in all the study population.

Persistence of reduced serum concentrations of vitamin E, may cause severe long lasting health consequences for children, such as impaired cognitive development [45]. Therefore, the results of the current study can be an alarming signal to pay attention for the importance of checking vitamin E level at birth.

An Algerian research determined that appropriate for gestational age term neonates showed umbilical cord vitamin E levels (528.5 µg/dL) which were higher than small for gestational age neonates (201.7 µg/dL) [46]. The previous study comes in parallel with our results.

Compatible with our results, there are information that AGA term neonates show greater vitamin E concentrations than those who are SGA [47]. Moreover, it was found that the more the birth weight, the more the vitamin E cord concentrations irrespective of the age of gestation at birth [9, 11]; indicating a potential correlation between fetal growth and vitamin E concentration. This is also in acceptance with our results as we detected significant positive correlation between weight for age Z scores and vitamin E cord blood levels.

Contradictory with our results, in a study done by Silva et al., vitamin E cord blood concentrations didn’t differ amongst all their studied neonates. They attributed that to the small sample size, which concealed the potential variations in vitamin E levels among their studied cohorts. Furthermore, they conducted their research on populations of Rio Grande do Norte, Brazil only. Silva et al., didn’t detect any association of cord blood concentrations of vitamin E with fetal growth, the decreased concentrations of vitamin E may have limited the results. However, they concluded that > 92% of the studied neonates presented reduced concentrations of vitamin E [11].

In the current study, the vitamin E levels in AGA neonates agreed with various studies [9, 47] and differed from others [48] possibly since the previous studies used citizens of variable nationalities. The researches conducted on Egyptians and Indians showed higher vitamin E serum levels than the cutoff point [9, 47, 49].

The majority of researchers described great proportions of neonates with reduced concentrations of vitamin E. A Tunisian study reported 55.5% of neonates at term had serum vitamin E levels lower than the cutoff point (301.7 µg/dL) [33].

Moreover, a study done to compare plasma vitamin E levels with selected pregnancy outcomes including; fetal growth between United States and Nigerian maternal–infant populations detected an inverse relationship between cord levels of vitamin E and 5-min APGAR scores in the United States of America (USA) group [15]. This is contradictory to our results as we couldn’t find any significant relation between vitamin E cord blood levels and Apgar scores. On the other hand, vitamin E cord blood concentrations among the cohort of Nigeria had insignificant correlation with APGAR scores which coincides with our results.

Furthermore, in the above mentioned study, they explored that in the subjects of United States, greater cord levels of vitamin E were correlated with neonatal length below the 10th percentile. This disagrees with our findings as there was no correlation between vitamin E level and neonatal length [15].

Vitamin D is essential for development, growth and the maintenance of the bone [50]. Sufficient vitamin D concentrations of the pregnant mother are needed, because the fetus relies completely on mother vitamin D. Vitamin D deficiency throughout pregnancy was connected with high possibility of pre-eclampsia and improper outcomes of pregnancy [51]. A 2019 Cochrane review meta-analysis detected that vitamin D administration during pregnancy diminished the risk of low birth weight [52].

The role of Vitamin D in growth of fetus in normal or complicated pregnancy has been investigated lately by many studies. It was detected that the placenta encompasses components involved in vitamin D signal transduction, i.e., vitamin D receptor (VDR) and CYP27B1 enzyme (1 alpha-hydroxylase) activating this vitamin. The active vitamin D, functioning via VDR and the cAMP/protein kinase signaling pathway, controls the expression and production of human chorionic gonadotropin in the syncytiotrophoblast and elevates the secretion of placental steroids [53].

Vitamin D is essential in glucose and insulin metabolic process too. Thus, it shares in confirming glucose accessibility for passage via the placenta and its consequent utilization by the fetus. Acting as a calcium homoeostasis and transportation controller, it affects growth of fetus, by influencing the skeletal muscles and bones development. Therefore, by this wide range of tasks taking place inside the growing fetus in pregnancy, the decreased weight of fetus as a result of its reduction is likely [54].

Deficiency of vitamin D which is prevalent in newborns, pediatric age group and throughout pregnancy is connected with high possibility of occurrence of gestational diabetes, preeclampsia, repeated abortions, LBW, and fetal IUGR [55, 56].

Estimation of 25(OH)D in blood is still the principle test for the evaluation of vitamin D state. The resulting values have to be evaluated in comparison to cut-offs which are built on medical hazards [57]. Deficiency of vitamin D has been determined as decreased 1 and 25-hydroxyvitamin D (25(OH) D) levels < 30 ng/mL [58].

Holick et al., [59] determined deficiency of vitamin D by a 25(OH)D concentration < 20 ng/mL (50 nmol/L), and insufficiency as a vitamin D level of 21–29 ng/mL (52.5–72.5 nmol/L). Conferring to Moon et al., [60] deficiency of vitamin D affects a great percentage of neonates at term reaching up to 97.4% of them.

A scarcity of information about the correlation between vitamin D cord blood level and pregnancy outcomes has been found. Considering the significance of various effects of vitamin D in newborns, vitamin D concentrations in cord blood were investigated in our study newborns, comparing its level in the IUGR neonates with AGA neonates and testing its association with birth weight.

Concerning vitamin D levels; our study showed that they were significantly lower in the cord blood of the IUGR group than the AGA group. Our study couldn’t detect any significant correlation of vitamin D cord blood concentrations with weight or length Z scores of all the study population. However, Head circumference Z scores showed significant positive correlations with vitamin D cord blood level at birth.

Many researches investigated the relation of vitamin D deficiency with IUGR occurrence [61]. There are evidences that the weight of fetus had positive correlation with maternal vitamin D concentrations [62].

A study done by Mahfod et al., detected that vitamin D serum level was significantly positively correlated with fetal biparietal diameter, abdominal circumference and weight [63]. Moreover, Tao et al., declared that deficient vitamin D in pregnancy influence significantly fetal bones growth and development, therefore, influencing the abdominal circumference, head circumference, importantly elevating the risk of SGA. The administration of vitamin D for more than two months during pregnancy lowered significantly the possibility of SGA in comparison to those lacking vitamin D administration (11.8% vs. 6.9%) [64]. Additionally, Alimohamadi et al. concluded that mothers with deficient vitamin D were 6 times at risk of IUGR than those with sufficient vitamin D concentrations. They proved that the incidence of IUGR could be predicted by assessing vitamin D concentrations at the beginning of pregnancy [65].

In contrast, Eggemoen et al. didn’t detect any association of vitamin D concentrations of mothers with anthropometric parameters of the newborns in a multiethnic cohort of pregnant mothers with deficient vitamin D [66]. A meta-analysis done by Zhao et al., didn’t detect any association between vitamin D sufficiency throughout pregnancy and IUGR. He concluded there was negative association between 25-hydroxyvitamin D levels of mothers and the possibility of low birth weight, while no association of vitamin D mother levels with Macrosomia (MA) or intrauterine growth restriction (IUGR) [67].

Interestingly, Jakubiec-Wisniewska et al., reported that administration of 2000 IU of vitamin D led to improvement in the cerebro-placental ratio (CPR) in fetuses having early growth restriction [68].

Studies showed that decreased vitamin D concentrations might play a role in reduction of normal growth of fetus, however, a number of researches delineated that the possibility of hypotrophy of fetus with deficient maternal vitamin D concentrations is multiple times greater [69, 70]. Conversely, not all researches approve an association of vitamin D with fetal growth [71].

The relation between decreased vitamin D concentrations and neonatal low birth weight was detected in normal and complicated pregnancy. In a study comprised 1198 USA mothers, it was shown that the neonatal birth weight was less in those with deficient vitamin D concentrations, and the possibility of the low birth weight diminished with elevation of maternal vitamin D concentrations to the higher border of the normal range [69].

Another research comprising more than 7000 mothers, delineated that fetal growth restriction in the third trimester further commonly happened in mothers with deficient vitamin D. Moreover, neonates had lesser weight, lesser head circumference, and body length. Deficiency of vitamin D < 20 ng/ml, increased the possibility of prematurity and intrauterine growth restriction significantly [72]. Moreover, Liu et al., postulated that neonatal serum vitamin D concentration was positively correlated with birth weight [34].

Prior researches have proved that dark skin, race, obesity, educational level, season, smoking, maternal vitamin D administration dose of < 15 µg/d will influence both maternal and neonatal level of vitamin D [73, 74]. In addition, it was accompanied with improvement of birth weight and length [75].

Singh et al., explained that 25(OH) D passes via the placental barrier during pregnancy. Based on the information that the placental decidua has receptors for vitamin D and expresses 1-∞-hydroxylase for the synthesis of 1, 25-dihydroxy vitamin D, shows possible associations between vitamin D and pregnancy outcomes. They concluded that low birth weight babies were more in vitamin D deficient than normal vitamin D mothers [76].

On the other hand, Zhu et al., concluded that, neonates having reduced or elevated vitamin D concentrations during fetal life were equally at high risk of growth retardation and greater vitamin D concentrations of fetus were not found to give better outcomes. An inverted U curve correlation of vitamin D levels of the neonates with intrauterine growth which does not imply causality [77]. While, Murmu et al., conducted a study in India which revealed strong negative correlation of the expected weight at birth calculated by ultrasonography during pregnancy at the start of the trial with the proportion of rise in neonatal weight at birth after administration of Vitamin D [6].

The contradictory outcomes of the above mentioned studies supporting the assumption that low levels of vitamin D leads to delayed linear growth of fetus and the other mentioned studies which didn’t support this hypothesis, might be because of differences in study methodologies comprising; the number of weeks of pregnancy at which vitamin D was assessed, vitamin D cut-off point, ethnicity of the studied subjects and genetic variability. Nevertheless, the variations of vitamin D concentrations could be partially referred to the differences in seasons of blood sampling and techniques of assessment. The interpretation of those results to clinical practice didn’t happen. Moreover, the majority of prior studies in this context have been conducted in developed countries. In addition, those studies had numerous drawbacks such as small size of sample, different descriptions for vitamin D state of fetus and insufficient confounders’ correction.

Vitamin status may influence fetal development. Newborns with intrauterine growth retardation (IUGR) may present with decreased Apgar score, require ventilation assistance and neonatal intensive care unit (NICU), brain insult with long-standing complications and chronic lung disease, in addition to the risk of death [78].

In this context, Kim et al., proved that the Apgar score of neonates with vitamin D minor insufficiency was greater than neonates with serious deficiency of vitamin D [79]. This is in contrast to our findings as insignificant correlation of cord blood concentrations of vitamin D with Apgar score was found.

In our work, although we detected positive correlation between birth weight and vitamin D level, this was statistically insignificant. This could be attributed to the small sample size. Furthermore, the variation between the findings of the present study and the above cited researches may be referred to variations in environment, season of sampling, socioeconomic level, and age of gestation.

The **main strengths** of our study is that it is the first one investigating the vitamin status of Egyptian neonates investigating three vitamins together not only in AGA but also in IUGR neonates. Second, when we divided the patients into 2 groups we tried to avoid confounding factors like (gestational age, twin, method of delivery, seasons of birth, maternal history, any pregnancy complications, socioeconomic level).

Nevertheless, **the limitations in this study** should also be admitted. **Firstly**, the small sample size. **Secondly**, we didn’t investigate maternal levels of these vitamins and the association between maternal and fetal levels. **Thirdly**, we measured the vitamin E in the form of α-tocopherol only while vitamin E normally presents in many tocopherol isomers (α, β, δ, and γ). We did this because, inspite that γ-tocopherol is the commonest isomer of vitamin E present in food, α-tocopherol has greater concentrations in human tissues, because of elevated breaking and elimination of γ-tocopherol, and elevated precipitation of α-tocopherol in cell membranes and lipoprotein–lipid bundles by α-tocopherol transfer protein (α-TTP) [80].

 Moreoer, considering that this study didn’t receive any financial support as it was self-funded and we couldn’t afford the cost of analyzing individual tocopherols, so we decided to measure α-tocopherol only based on that α-tocopherol level, particularly is connected with outcomes of pregnancy, like growth of fetus, has been approved [15].

**Fourthly**, we assessed the levels of vitamin D in the form of 25 (OH) D only, that is one of the vitamin D forms, while, alterations of vitamin D binding protein, 1,25(OH)2D, the CYP27 enzyme and vitamin D receptors expression may occur at birth, that can affect the relation of 25(OH)D with growth of fetus [81]. Therefore, upcoming studies of association between vitamin D and fetal growth retardation should consider other forms of vitamin D.

## Conclusions

Neonates with IUGR had significantly lower levels of Vitamin A, Vitamin E and Vitamin D than AGA neonates. Significant positive correlations of weight with vitamin A, and E cord blood levels was found. While neonatal length significantly positively correlated only with vitamin A level. However, we couldn’t detect significant associations between vitamin D level and neonatal birth weight or length. Thus, because of the implications of growth restriction in neonates, it is important to explore the elements which might cause vitamins deficiency and follow up of children’s nutritional status.

Our results lay emphasis on the significance of nutritional supplementary policies to prevent maternal and child dyads deficiency of these vitamins (A, E & D). Boosting breastfeeding during the 1st 6 months of infancy and supporting satisfactory dietary consumptions of these vitamins in pregnant and lactating mothers are important actions in managing this dietary insufficiency.

## Data Availability

Data are available from the corresponding author upon reasonable request.

## Abbreviations

AGA: Appropriate for Gestational Age
BMI: Body Mass Index
CS: Caesarean Section
ELISA: Enzyme-linked Immuno-Sorbent Assay
FAO: Food and Agriculture Organization
GA: Gestational Age
HPLC: High performance liquid chromatography
IUGR: Intrauterine Growth Retardation
LBW: Low Birth Weight
LMP: Last Menstrual Period
NICU: Neonatal Intensive Care Unit
SGA: Small for Gestational Age
U/S: Ultrasound
USA: United States of America
WHO: World Health Organization
